# Supplementary material for: Gestational weight gain at the national, regional, and income group levels based on 234 national household surveys from 70 low-income and middle-income countries
Source: PLOS Glob Public Health. 2024 Sep 4;4(9):e0003484. doi: 10.1371/journal.pgph.0003484 (PMC11373806; doi:10.1371/journal.pgph.0003484)
Supplement: S1 Appendix — (DOCX) [file pgph.0003484.s001.docx]

**S1 Appendix - Description of the analytical framework**

1. **Estimating GWG from data nationally representative survey data**
   1. The cross-sectional gestational weight measures for women in the second and third trimesters of pregnancy were regressed on the corresponding gestational months using an ordinary least squares regression model, accounting for cluster, stratification and sampling weights. Control variables were maternal age, years of education, number of children ever born, urban or rural residence, and quintiles of household wealth. The β coefficient for the survey-specific models represents the average monthly weight gain in the second and third trimesters.
      $Gestational Weighti = \alpha+ \beta\times Gestational Monthi + [Control Variables]$
   2. The mean total GWG for the entire pregnancy was calculated by multiplying the beta coefficient by the weight gain during the first trimester relative to the second and third trimesters (as a percentage) and was extracted from the GWG charts of the LifeCycle Consortium and by a constant that represents the duration of a full-term pregnancy in months.
      $(1+P)\times6.5\times\beta$
   3. To calculate the uncertainty due to sampling variability, we computed the total GWG 95% confidence interval as $(1+P)\times6.5\times(\beta\pm1.96\times{SE}_{\beta})$, where SE_β_ represents the SE estimate of β.
2. **Mixed-effect hierarchical model**
   1. The two-level hierarchical model used the estimated total GWG as the dependent variable and year and country-level covariates as the independent variables.
   2. The model included a fixed intercept and fixed effects for year, geographical superregion, and country-level predictors.
   3. The model included country-specific random intercepts and random effects for the survey year.
   4. Models with only a linear term for survey year and with additional restricted cubic splines terms for survey year using different numbers (3, 4, or 5) and locations of knots were compared, and the one with the smallest value of the Bayesian information criterion (BIC) was selected.
   5. Geographical super regions were those used in the Global Burden of Disease (GBD) Study: (1) Sub-Saharan Africa; (2) Latin America and the Caribbean; (3) Southeast Asia, East Asia, and Oceania; (4) South Asia; (5) North Africa and the Middle East; and (6) Central Europe, Eastern Europe, and Central Asia.
3. **Selection of country-level covariates**
   1. The potential country-level covariates included:

- neonatal mortality rate,
- low birthweight rate,
- proportion of women receiving four or more antenatal care visits,
- caesarean section rate,
- mean adult female BMI,
- gross domestic product (GDP) per capita,
- Gini index, Human Development Index (HDI),
- total fertility rate,
- adolescent fertility rate,
- percentage of adolescents aged 15–19 years out of all women of reproductive age, and
- adult female literacy rate.
  1. These covariates were predictors that may be associated with mean GWG at the population level. It is of note that the purpose of the hierarchical models was to build prediction instead of conducting causal estimation. Therefore, the country-level covariates were not necessarily causal determinants of GWG at the individual level, and some covariates may not temporarily precede GWG.
  2. We obtained data on the country-level predictors from publicly available databases and excluded candidate covariates with more than 30% of missing values for the years when gestational weight data were available in the surveys (low birthweight rate, proportion of women receiving four or more antenatal care visits, cesarean section rate, Gini index, and adult female literacy rate).
  3. Correlations between the country-level covariates were tested by using the variance inflation factor (VIF), and the HDI, which had a VIF greater than 10, was removed from the model.
  4. We then used the BIC to further guide variable selection and avoid overfitting. One predictor at a time was removed from the full model, starting with the one presenting the largest BIC in the univariate analysis. When the exclusion of a given predictor resulted in a smaller BIC, it was dropped from the model; otherwise, the predictor was retained in
