# Supplementary material for: Gestational weight gain at the national, regional, and income group levels based on 234 national household surveys from 70 low-income and middle-income countries
Source: PLOS Glob Public Health. 2024 Sep 4;4(9):e0003484. doi: 10.1371/journal.pgph.0003484 (PMC11373806; doi:10.1371/journal.pgph.0003484)
Supplement: S1 Table — (DOCX) [file pgph.0003484.s002.docx]

**S1 Table – List of surveys (country and year) included in the analysis and the corresponding super-region, region, and income group.**

| **Country** | **Year** | **Region** | **Sub region** | **Country Income Level** |
| --- | --- | --- | --- | --- |
| Albania | 2017 | Central Europe, Eastern Europe, and Central Asia | Central Europe | Upper-middle-income |
| Albania | 2008 | Central Europe, Eastern Europe, and Central Asia | Central Europe | Upper-middle-income |
| Armenia | 2015 | Central Europe, Eastern Europe, and Central Asia | Central Asia | Upper-middle-income |
| Armenia | 2005 | Central Europe, Eastern Europe, and Central Asia | Central Asia | Upper-middle-income |
| Armenia | 2000 | Central Europe, Eastern Europe, and Central Asia | Central Asia | Upper-middle-income |
| Azerbaijan | 2006 | Central Europe, Eastern Europe, and Central Asia | Central Asia | Upper-middle-income |
| Burundi | 2016 | Sub-Saharan Africa | Eastern Sub-Saharan Africa | Low-income |
| Burundi | 2010 | Sub-Saharan Africa | Eastern Sub-Saharan Africa | Low-income |
| Benin | 2017 | Sub-Saharan Africa | Western Sub-Saharan Africa | Low-income |
| Benin | 1996 | Sub-Saharan Africa | Western Sub-Saharan Africa | Low-income |
| Benin | 2001 | Sub-Saharan Africa | Western Sub-Saharan Africa | Low-income |
| Benin | 2006 | Sub-Saharan Africa | Western Sub-Saharan Africa | Low-income |
| Benin | 2011 | Sub-Saharan Africa | Western Sub-Saharan Africa | Low-income |
| Burkina Faso | 2010 | Sub-Saharan Africa | Western Sub-Saharan Africa | Low-income |
| Burkina Faso | 2021 | Sub-Saharan Africa | Western Sub-Saharan Africa | Low-income |
| Burkina Faso | 1993 | Sub-Saharan Africa | Western Sub-Saharan Africa | Low-income |
| Burkina Faso | 2003 | Sub-Saharan Africa | Western Sub-Saharan Africa | Low-income |
| Burkina Faso | 1998 | Sub-Saharan Africa | Western Sub-Saharan Africa | Low-income |
| Bangladesh | 2014 | South Asia | South Asia | Lower-middle-income |
| Bangladesh | 1996 | South Asia | South Asia | Lower-middle-income |
| Bangladesh | 2017 | South Asia | South Asia | Lower-middle-income |
| Bangladesh | 2004 | South Asia | South Asia | Lower-middle-income |
| Bangladesh | 1999 | South Asia | South Asia | Lower-middle-income |
| Bangladesh | 2011 | South Asia | South Asia | Lower-middle-income |
| Bangladesh | 2007 | South Asia | South Asia | Lower-middle-income |
| Bolivia | 1998 | Latin America and the Caribbean | Andean Latin America | Lower-middle-income |
| Bolivia | 2008 | Latin America and the Caribbean | Andean Latin America | Lower-middle-income |
| Bolivia | 1994 | Latin America and the Caribbean | Andean Latin America | Lower-middle-income |
| Bolivia | 2016 | Latin America and the Caribbean | Andean Latin America | Lower-middle-income |
| Bolivia | 2003 | Latin America and the Caribbean | Andean Latin America | Lower-middle-income |
| Brazil | 1996 | Latin America and the Caribbean | Tropical Latin America | Upper-middle-income |
| Brazil | 2006 | Latin America and the Caribbean | Tropical Latin America | Upper-middle-income |
| Central African Republic | 1994 | Sub-Saharan Africa | Central Sub-Saharan Africa | Low-income |
| Cote d'Ivoire | 2011 | Sub-Saharan Africa | Western Sub-Saharan Africa | Lower-middle-income |
| Cote d'Ivoire | 2021 | Sub-Saharan Africa | Western Sub-Saharan Africa | Lower-middle-income |
| Cote d'Ivoire | 1998 | Sub-Saharan Africa | Western Sub-Saharan Africa | Lower-middle-income |
| Cote d'Ivoire | 1994 | Sub-Saharan Africa | Western Sub-Saharan Africa | Lower-middle-income |
| Cameroon | 2018 | Sub-Saharan Africa | Western Sub-Saharan Africa | Lower-middle-income |
| Cameroon | 2004 | Sub-Saharan Africa | Western Sub-Saharan Africa | Lower-middle-income |
| Cameroon | 1998 | Sub-Saharan Africa | Western Sub-Saharan Africa | Lower-middle-income |
| Cameroon | 2011 | Sub-Saharan Africa | Western Sub-Saharan Africa | Lower-middle-income |
| Democratic Republic of Congo | 2013 | Sub-Saharan Africa | Central Sub-Saharan Africa | Low-income |
| Democratic Republic of Congo | 2007 | Sub-Saharan Africa | Central Sub-Saharan Africa | Low-income |
| Congo | 2005 | Sub-Saharan Africa | Central Sub-Saharan Africa | Lower-middle-income |
| Congo | 2011 | Sub-Saharan Africa | Central Sub-Saharan Africa | Lower-middle-income |
| Colombia | 2010 | Latin America and the Caribbean | Central Latin America | Upper-middle-income |
| Colombia | 2005 | Latin America and the Caribbean | Central Latin America | Upper-middle-income |
| Colombia | 1995 | Latin America and the Caribbean | Central Latin America | Upper-middle-income |
| Colombia | 2000 | Latin America and the Caribbean | Central Latin America | Upper-middle-income |
| Comoros | 1996 | Sub-Saharan Africa | Eastern Sub-Saharan Africa | Lower-middle-income |
| Comoros | 2012 | Sub-Saharan Africa | Eastern Sub-Saharan Africa | Lower-middle-income |
| Dominican Republic | 2013 | Latin America and the Caribbean | Caribbean | Upper-middle-income |
| Dominican Republic | 1996 | Latin America and the Caribbean | Caribbean | Upper-middle-income |
| Dominican Republic | 1991 | Latin America and the Caribbean | Caribbean | Upper-middle-income |
| Ecuador | 2018 | Latin America and the Caribbean | Andean Latin America | Upper-middle-income |
| Egypt | 2008 | North Africa and the Middle East | North Africa and the Middle East | Lower-middle-income |
| Egypt | 2005 | North Africa and the Middle East | North Africa and the Middle East | Lower-middle-income |
| Egypt | 2014 | North Africa and the Middle East | North Africa and the Middle East | Lower-middle-income |
| Egypt | 2003 | North Africa and the Middle East | North Africa and the Middle East | Lower-middle-income |
| Egypt | 1995 | North Africa and the Middle East | North Africa and the Middle East | Lower-middle-income |
| Egypt | 1992 | North Africa and the Middle East | North Africa and the Middle East | Lower-middle-income |
| Egypt | 2000 | North Africa and the Middle East | North Africa and the Middle East | Lower-middle-income |
| Ethiopia | 2005 | Sub-Saharan Africa | Eastern Sub-Saharan Africa | Low-income |
| Ethiopia | 2011 | Sub-Saharan Africa | Eastern Sub-Saharan Africa | Low-income |
| Ethiopia | 2000 | Sub-Saharan Africa | Eastern Sub-Saharan Africa | Low-income |
| Ethiopia | 2016 | Sub-Saharan Africa | Eastern Sub-Saharan Africa | Low-income |
| Gabon | 2012 | Sub-Saharan Africa | Central Sub-Saharan Africa | Upper-middle-income |
| Gabon | 2019 | Sub-Saharan Africa | Central Sub-Saharan Africa | Upper-middle-income |
| Gabon | 2000 | Sub-Saharan Africa | Central Sub-Saharan Africa | Upper-middle-income |
| Ghana | 1993 | Sub-Saharan Africa | Western Sub-Saharan Africa | Lower-middle-income |
| Ghana | 2014 | Sub-Saharan Africa | Western Sub-Saharan Africa | Lower-middle-income |
| Ghana | 2003 | Sub-Saharan Africa | Western Sub-Saharan Africa | Lower-middle-income |
| Ghana | 2008 | Sub-Saharan Africa | Western Sub-Saharan Africa | Lower-middle-income |
| Ghana | 1998 | Sub-Saharan Africa | Western Sub-Saharan Africa | Lower-middle-income |
| Guinea | 2012 | Sub-Saharan Africa | Western Sub-Saharan Africa | Low-income |
| Guinea | 1999 | Sub-Saharan Africa | Western Sub-Saharan Africa | Low-income |
| Guinea | 2005 | Sub-Saharan Africa | Western Sub-Saharan Africa | Low-income |
| Guinea | 2018 | Sub-Saharan Africa | Western Sub-Saharan Africa | Low-income |
| The Gambia | 2013 | Sub-Saharan Africa | Western Sub-Saharan Africa | Low-income |
| The Gambia | 2019 | Sub-Saharan Africa | Western Sub-Saharan Africa | Low-income |
| Guatemala | 1998 | Latin America and the Caribbean | Central Latin America | Upper-middle-income |
| Guatemala | 1995 | Latin America and the Caribbean | Central Latin America | Upper-middle-income |
| Guyana | 2009 | Latin America and the Caribbean | Caribbean | Upper-middle-income |
| Honduras | 2005 | Latin America and the Caribbean | Central Latin America | Lower-middle-income |
| Honduras | 2011 | Latin America and the Caribbean | Central Latin America | Lower-middle-income |
| Haiti | 2016 | Latin America and the Caribbean | Caribbean | Low-income |
| Haiti | 2005 | Latin America and the Caribbean | Caribbean | Low-income |
| Haiti | 1994 | Latin America and the Caribbean | Caribbean | Low-income |
| Haiti | 2000 | Latin America and the Caribbean | Caribbean | Low-income |
| Haiti | 2012 | Latin America and the Caribbean | Caribbean | Low-income |
| India | 2015 | South Asia | South Asia | Lower-middle-income |
| India | 2019 | South Asia | South Asia | Lower-middle-income |
| India | 1998 | South Asia | South Asia | Lower-middle-income |
| India | 2005 | South Asia | South Asia | Lower-middle-income |
| Jordan | 2017 | North Africa and the Middle East | North Africa and the Middle East | Upper-middle-income |
| Jordan | 2009 | North Africa and the Middle East | North Africa and the Middle East | Upper-middle-income |
| Jordan | 1997 | North Africa and the Middle East | North Africa and the Middle East | Upper-middle-income |
| Jordan | 2002 | North Africa and the Middle East | North Africa and the Middle East | Upper-middle-income |
| Jordan | 2012 | North Africa and the Middle East | North Africa and the Middle East | Upper-middle-income |
| Jordan | 2007 | North Africa and the Middle East | North Africa and the Middle East | Upper-middle-income |
| Kazakhstan | 1995 | Central Europe, Eastern Europe, and Central Asia | Central Asia | Upper-middle-income |
| Kazakhstan | 1999 | Central Europe, Eastern Europe, and Central Asia | Central Asia | Upper-middle-income |
| Kenya | 1998 | Sub-Saharan Africa | Eastern Sub-Saharan Africa | Lower-middle-income |
| Kenya | 2003 | Sub-Saharan Africa | Eastern Sub-Saharan Africa | Lower-middle-income |
| Kenya | 2022 | Sub-Saharan Africa | Eastern Sub-Saharan Africa | Lower-middle-income |
| Kenya | 2008 | Sub-Saharan Africa | Eastern Sub-Saharan Africa | Lower-middle-income |
| Kenya | 2014 | Sub-Saharan Africa | Eastern Sub-Saharan Africa | Lower-middle-income |
| Kenya | 1993 | Sub-Saharan Africa | Eastern Sub-Saharan Africa | Lower-middle-income |
| Kyrgyz Republic | 1997 | Central Europe, Eastern Europe, and Central Asia | Central Asia | Lower-middle-income |
| Kyrgyz Republic | 2012 | Central Europe, Eastern Europe, and Central Asia | Central Asia | Lower-middle-income |
| Cambodia | 2005 | Southeast Asia, East Asia, and Oceania | Southeast Asia | Lower-middle-income |
| Cambodia | 2000 | Southeast Asia, East Asia, and Oceania | Southeast Asia | Lower-middle-income |
| Cambodia | 2014 | Southeast Asia, East Asia, and Oceania | Southeast Asia | Lower-middle-income |
| Cambodia | 2010 | Southeast Asia, East Asia, and Oceania | Southeast Asia | Lower-middle-income |
| Cambodia | 2021 | Southeast Asia, East Asia, and Oceania | Southeast Asia | Lower-middle-income |
| Liberia | 2013 | Sub-Saharan Africa | Western Sub-Saharan Africa | Low-income |
| Liberia | 2007 | Sub-Saharan Africa | Western Sub-Saharan Africa | Low-income |
| Liberia | 2019 | Sub-Saharan Africa | Western Sub-Saharan Africa | Low-income |
| Lesotho | 2009 | Sub-Saharan Africa | Southern Sub-Saharan Africa | Lower-middle-income |
| Lesotho | 2014 | Sub-Saharan Africa | Southern Sub-Saharan Africa | Lower-middle-income |
| Lesotho | 2004 | Sub-Saharan Africa | Southern Sub-Saharan Africa | Lower-middle-income |
| Morocco | 1992 | North Africa and the Middle East | North Africa and the Middle East | Lower-middle-income |
| Morocco | 2003 | North Africa and the Middle East | North Africa and the Middle East | Lower-middle-income |
| Moldova | 2005 | Central Europe, Eastern Europe, and Central Asia | Eastern Europe | Lower-middle-income |
| Madagascar | 2021 | Sub-Saharan Africa | Eastern Sub-Saharan Africa | Low-income |
| Madagascar | 2003 | Sub-Saharan Africa | Eastern Sub-Saharan Africa | Low-income |
| Madagascar | 1997 | Sub-Saharan Africa | Eastern Sub-Saharan Africa | Low-income |
| Madagascar | 2008 | Sub-Saharan Africa | Eastern Sub-Saharan Africa | Low-income |
| Maldives | 2016 | Southeast Asia, East Asia, and Oceania | Southeast Asia | Upper-middle-income |
| Maldives | 2009 | Southeast Asia, East Asia, and Oceania | Southeast Asia | Upper-middle-income |
| Mexico | 2018 | Latin America and the Caribbean | Central Latin America | Upper-middle-income |
| Mali | 2018 | Sub-Saharan Africa | Western Sub-Saharan Africa | Low-income |
| Mali | 2006 | Sub-Saharan Africa | Western Sub-Saharan Africa | Low-income |
| Mali | 1995 | Sub-Saharan Africa | Western Sub-Saharan Africa | Low-income |
| Mali | 2001 | Sub-Saharan Africa | Western Sub-Saharan Africa | Low-income |
| Mali | 2012 | Sub-Saharan Africa | Western Sub-Saharan Africa | Low-income |
| Myanmar | 2015 | Southeast Asia, East Asia, and Oceania | Southeast Asia | Lower-middle-income |
| Mozambique | 1997 | Sub-Saharan Africa | Eastern Sub-Saharan Africa | Low-income |
| Mozambique | 2011 | Sub-Saharan Africa | Eastern Sub-Saharan Africa | Low-income |
| Mozambique | 2003 | Sub-Saharan Africa | Eastern Sub-Saharan Africa | Low-income |
| Mauritania | 2019 | Sub-Saharan Africa | Western Sub-Saharan Africa | Lower-middle-income |
| Malawi | 2015 | Sub-Saharan Africa | Eastern Sub-Saharan Africa | Low-income |
| Malawi | 2004 | Sub-Saharan Africa | Eastern Sub-Saharan Africa | Low-income |
| Malawi | 2000 | Sub-Saharan Africa | Eastern Sub-Saharan Africa | Low-income |
| Malawi | 2010 | Sub-Saharan Africa | Eastern Sub-Saharan Africa | Low-income |
| Malawi | 1992 | Sub-Saharan Africa | Eastern Sub-Saharan Africa | Low-income |
| Namibia | 2006 | Sub-Saharan Africa | Southern Sub-Saharan Africa | Upper-middle-income |
| Namibia | 1992 | Sub-Saharan Africa | Southern Sub-Saharan Africa | Upper-middle-income |
| Namibia | 2013 | Sub-Saharan Africa | Southern Sub-Saharan Africa | Upper-middle-income |
| Niger | 1992 | Sub-Saharan Africa | Western Sub-Saharan Africa | Low-income |
| Niger | 2012 | Sub-Saharan Africa | Western Sub-Saharan Africa | Low-income |
| Niger | 1998 | Sub-Saharan Africa | Western Sub-Saharan Africa | Low-income |
| Niger | 2006 | Sub-Saharan Africa | Western Sub-Saharan Africa | Low-income |
| Nigeria | 2003 | Sub-Saharan Africa | Western Sub-Saharan Africa | Lower-middle-income |
| Nigeria | 2008 | Sub-Saharan Africa | Western Sub-Saharan Africa | Lower-middle-income |
| Nigeria | 2018 | Sub-Saharan Africa | Western Sub-Saharan Africa | Lower-middle-income |
| Nigeria | 2013 | Sub-Saharan Africa | Western Sub-Saharan Africa | Lower-middle-income |
| Nicaragua | 2001 | Latin America and the Caribbean | Central Latin America | Lower-middle-income |
| Nicaragua | 1998 | Latin America and the Caribbean | Central Latin America | Lower-middle-income |
| Nepal | 2006 | South Asia | South Asia | Low-income |
| Nepal | 2001 | South Asia | South Asia | Low-income |
| Nepal | 2022 | South Asia | South Asia | Low-income |
| Nepal | 1996 | South Asia | South Asia | Low-income |
| Nepal | 2016 | South Asia | South Asia | Low-income |
| Nepal | 2011 | South Asia | South Asia | Low-income |
| Pakistan | 2012 | South Asia | South Asia | Lower-middle-income |
| Pakistan | 2017 | South Asia | South Asia | Lower-middle-income |
| Peru | 1996 | Latin America and the Caribbean | Andean Latin America | Upper-middle-income |
| Peru | 2017 | Latin America and the Caribbean | Andean Latin America | Upper-middle-income |
| Peru | 2020 | Latin America and the Caribbean | Andean Latin America | Upper-middle-income |
| Peru | 2018 | Latin America and the Caribbean | Andean Latin America | Upper-middle-income |
| Peru | 2009 | Latin America and the Caribbean | Andean Latin America | Upper-middle-income |
| Peru | 2010 | Latin America and the Caribbean | Andean Latin America | Upper-middle-income |
| Peru | 1991 | Latin America and the Caribbean | Andean Latin America | Upper-middle-income |
| Peru | 2016 | Latin America and the Caribbean | Andean Latin America | Upper-middle-income |
| Peru | 2011 | Latin America and the Caribbean | Andean Latin America | Upper-middle-income |
| Peru | 2012 | Latin America and the Caribbean | Andean Latin America | Upper-middle-income |
| Peru | 2019 | Latin America and the Caribbean | Andean Latin America | Upper-middle-income |
| Peru | 2013 | Latin America and the Caribbean | Andean Latin America | Upper-middle-income |
| Peru | 2014 | Latin America and the Caribbean | Andean Latin America | Upper-middle-income |
| Peru | 2000 | Latin America and the Caribbean | Andean Latin America | Upper-middle-income |
| Peru | 2015 | Latin America and the Caribbean | Andean Latin America | Upper-middle-income |
| Peru | 2006 | Latin America and the Caribbean | Andean Latin America | Upper-middle-income |
| Rwanda | 2014 | Sub-Saharan Africa | Eastern Sub-Saharan Africa | Low-income |
| Rwanda | 2005 | Sub-Saharan Africa | Eastern Sub-Saharan Africa | Low-income |
| Rwanda | 2019 | Sub-Saharan Africa | Eastern Sub-Saharan Africa | Low-income |
| Rwanda | 2010 | Sub-Saharan Africa | Eastern Sub-Saharan Africa | Low-income |
| Rwanda | 2000 | Sub-Saharan Africa | Eastern Sub-Saharan Africa | Low-income |
| Senegal | 1992 | Sub-Saharan Africa | Western Sub-Saharan Africa | Lower-middle-income |
| Senegal | 2010 | Sub-Saharan Africa | Western Sub-Saharan Africa | Lower-middle-income |
| Senegal | 2005 | Sub-Saharan Africa | Western Sub-Saharan Africa | Lower-middle-income |
| Sierra Leone | 2008 | Sub-Saharan Africa | Western Sub-Saharan Africa | Low-income |
| Sierra Leone | 2013 | Sub-Saharan Africa | Western Sub-Saharan Africa | Low-income |
| Sierra Leone | 2019 | Sub-Saharan Africa | Western Sub-Saharan Africa | Low-income |
| Sao Tome and Principe | 2008 | Sub-Saharan Africa | Western Sub-Saharan Africa | Lower-middle-income |
| Eswatini | 2006 | Sub-Saharan Africa | Southern Sub-Saharan Africa | Lower-middle-income |
| Chad | 2014 | Sub-Saharan Africa | Western Sub-Saharan Africa | Low-income |
| Chad | 2004 | Sub-Saharan Africa | Western Sub-Saharan Africa | Low-income |
| Chad | 1996 | Sub-Saharan Africa | Western Sub-Saharan Africa | Low-income |
| Togo | 1998 | Sub-Saharan Africa | Western Sub-Saharan Africa | Low-income |
| Togo | 2013 | Sub-Saharan Africa | Western Sub-Saharan Africa | Low-income |
| Tajikistan | 2012 | Central Europe, Eastern Europe, and Central Asia | Central Asia | Low-income |
| Tajikistan | 2017 | Central Europe, Eastern Europe, and Central Asia | Central Asia | Low-income |
| Timor-Leste | 2009 | Southeast Asia, East Asia, and Oceania | Southeast Asia | Lower-middle-income |
| Timor-Leste | 2016 | Southeast Asia, East Asia, and Oceania | Southeast Asia | Lower-middle-income |
| Turkey | 2008 | North Africa and the Middle East | North Africa and the Middle East | Upper-middle-income |
| Turkey | 2018 | North Africa and the Middle East | North Africa and the Middle East | Upper-middle-income |
| Turkey | 1998 | North Africa and the Middle East | North Africa and the Middle East | Upper-middle-income |
| Turkey | 2003 | North Africa and the Middle East | North Africa and the Middle East | Upper-middle-income |
| Turkey | 1993 | North Africa and the Middle East | North Africa and the Middle East | Upper-middle-income |
| Turkey | 2013 | North Africa and the Middle East | North Africa and the Middle East | Upper-middle-income |
| Tanzania | 2015 | Sub-Saharan Africa | Eastern Sub-Saharan Africa | Low-income |
| Tanzania | 1991 | Sub-Saharan Africa | Eastern Sub-Saharan Africa | Low-income |
| Tanzania | 1996 | Sub-Saharan Africa | Eastern Sub-Saharan Africa | Low-income |
| Tanzania | 2010 | Sub-Saharan Africa | Eastern Sub-Saharan Africa | Low-income |
| Tanzania | 2004 | Sub-Saharan Africa | Eastern Sub-Saharan Africa | Low-income |
| Uganda | 2000 | Sub-Saharan Africa | Eastern Sub-Saharan Africa | Low-income |
| Uganda | 2011 | Sub-Saharan Africa | Eastern Sub-Saharan Africa | Low-income |
| Uganda | 1995 | Sub-Saharan Africa | Eastern Sub-Saharan Africa | Low-income |
| Uganda | 2016 | Sub-Saharan Africa | Eastern Sub-Saharan Africa | Low-income |
| Uganda | 2006 | Sub-Saharan Africa | Eastern Sub-Saharan Africa | Low-income |
| Uzbekistan | 1996 | Central Europe, Eastern Europe, and Central Asia | Central Asia | Lower-middle-income |
| Yemen | 2013 | North Africa and the Middle East | North Africa and the Middle East | Low-income |
| South Africa | 2016 | Sub-Saharan Africa | Southern Sub-Saharan Africa | Upper-middle-income |
| Zambia | 1992 | Sub-Saharan Africa | Eastern Sub-Saharan Africa | Lower-middle-income |
| Zambia | 2007 | Sub-Saharan Africa | Eastern Sub-Saharan Africa | Lower-middle-income |
| Zambia | 2001 | Sub-Saharan Africa | Eastern Sub-Saharan Africa | Lower-middle-income |
| Zambia | 2013 | Sub-Saharan Africa | Eastern Sub-Saharan Africa | Lower-middle-income |
| Zambia | 1996 | Sub-Saharan Africa | Eastern Sub-Saharan Africa | Lower-middle-income |
| Zimbabwe | 1999 | Sub-Saharan Africa | Southern Sub-Saharan Africa | Lower-middle-income |
| Zimbabwe | 2005 | Sub-Saharan Africa | Southern Sub-Saharan Africa | Lower-middle-income |
| Zimbabwe | 1994 | Sub-Saharan Africa | Southern Sub-Saharan Africa | Lower-middle-income |
| Zimbabwe | 2010 | Sub-Saharan Africa | Southern Sub-Saharan Africa | Lower-middle-income |
| Zimbabwe | 2015 | Sub-Saharan Africa | Southern Sub-Saharan Africa | Lower-middle-income |

Note: shadowed cells indicate the 28 surveys not included in the analysis by Wang et al (2020).
