# Supplementary material for: Gestational weight gain at the national, regional, and income group levels based on 234 national household surveys from 70 low-income and middle-income countries
Source: PLOS Glob Public Health. 2024 Sep 4;4(9):e0003484. doi: 10.1371/journal.pgph.0003484 (PMC11373806; doi:10.1371/journal.pgph.0003484)
Supplement: S2 Table — (DOCX) [file pgph.0003484.s003.docx]

**S2 Table –** **Country-specific gestational weight gain (GWG) estimates for the year 2020 derived from hierarchical modeling and the corresponding 95% uncertainty range limits.**

| **Country** | **Region** | **Sub Region** | **Country Income Level** | **GWG Estimate (kg)** | **95% Uncertainty Range** | |
| --- | --- | --- | --- | --- | --- | --- |
|  |  |  |  |  | **Lower Bound** | **Upper Bound** |
| Afghanistan | North Africa and the Middle East | North Africa and the Middle East | Low-income | 5.0 | 1.8 | 8.2 |
| Albania | Central Europe, Eastern Europe, and Central Asia | Central Europe | Upper-middle-income | 9.8 | 5.7 | 13.9 |
| Algeria | North Africa and the Middle East | North Africa and the Middle East | Upper-middle-income | 7.4 | 4.9 | 10.0 |
| Angola | Sub‐Saharan Africa | Central Sub‐Saharan Africa | Lower-middle-income | 5.7 | 3.4 | 7.9 |
| Argentina | Latin America and the Caribbean | Tropical Latin America | Upper-middle-income | 9.9 | 7.6 | 12.3 |
| Armenia | Central Europe, Eastern Europe, and Central Asia | Central Asia | Upper-middle-income | 11.7 | 6.9 | 16.5 |
| Azerbaijan | Central Europe, Eastern Europe, and Central Asia | Central Asia | Upper-middle-income | 10.4 | 6.8 | 14.0 |
| Bangladesh | South Asia | South Asia | Lower-middle-income | 8.7 | 5.0 | 12.4 |
| Belarus | Central Europe, Eastern Europe, and Central Asia | Eastern Europe | Upper-middle-income | 12.9 | 9.7 | 16.0 |
| Belize | Latin America and the Caribbean | Caribbean | Upper-middle-income | 8.5 | 5.1 | 11.9 |
| Benin | Sub‐Saharan Africa | Western Sub‐Saharan Africa | Low-income | 7.6 | 1.6 | 13.6 |
| Bhutan | South Asia | South Asia | Lower-middle-income | 8.6 | 6.0 | 11.3 |
| Bolivia | Latin America and the Caribbean | Andean Latin America | Lower-middle-income | 9.7 | 5.0 | 14.3 |
| Bosnia and Herzegovina | Central Europe, Eastern Europe, and Central Asia | Central Europe | Upper-middle-income | 13.5 | 10.3 | 16.7 |
| Botswana | Sub‐Saharan Africa | Southern Sub‐Saharan Africa | Upper-middle-income | 8.8 | 6.3 | 11.3 |
| Brazil | Latin America and the Caribbean | Tropical Latin America | Upper-middle-income | 13.2 | 10.5 | 16.0 |
| Bulgaria | Central Europe, Eastern Europe, and Central Asia | Central Europe | Upper-middle-income | 13.4 | 9.9 | 16.8 |
| Burkina Faso | Sub‐Saharan Africa | Western Sub‐Saharan Africa | Low-income | 6.6 | 2.7 | 10.4 |
| Burundi | Sub‐Saharan Africa | Eastern Sub‐Saharan Africa | Low-income | 6.5 | 3.4 | 9.7 |
| Cambodia | Southeast Asia, East Asia, and Oceania | Southeast Asia | Lower-middle-income | 8.6 | 5.3 | 11.9 |
| Cameroon | Sub‐Saharan Africa | Western Sub‐Saharan Africa | Lower-middle-income | 7.2 | 2.4 | 11.9 |
| Cape Verde | Sub‐Saharan Africa | Western Sub‐Saharan Africa | Lower-middle-income | 9.8 | 7.1 | 12.5 |
| Central African Republic | Sub‐Saharan Africa | Central Sub‐Saharan Africa | Low-income | 2.6 | -2.9 | 8.2 |
| Chad | Sub‐Saharan Africa | Western Sub‐Saharan Africa | Low-income | 5.2 | 1.7 | 8.7 |
| China | Southeast Asia, East Asia, and Oceania | East Asia | Upper-middle-income | 10.1 | 7.5 | 12.8 |
| Colombia | Latin America and the Caribbean | Central Latin America | Upper-middle-income | 11.6 | 7.3 | 15.9 |
| Comoros | Sub‐Saharan Africa | Eastern Sub‐Saharan Africa | Lower-middle-income | 9.7 | 4.3 | 15.0 |
| Congo | Sub‐Saharan Africa | Central Sub‐Saharan Africa | Lower-middle-income | 5.1 | -5.5 | 15.6 |
| Congo Democratic Republic | Sub‐Saharan Africa | Central Sub‐Saharan Africa | Low-income | 4.6 | 2.5 | 6.8 |
| Costa Rica | Latin America and the Caribbean | Central Latin America | Upper-middle-income | 10.5 | 8.1 | 12.8 |
| Cote d'Ivoire | Sub‐Saharan Africa | Western Sub‐Saharan Africa | Lower-middle-income | 6.9 | 0.9 | 12.8 |
| Cuba | Latin America and the Caribbean | Caribbean | Upper-middle-income | 11.0 | 8.8 | 13.3 |
| Dem Peoples Republic of Korea | Southeast Asia, East Asia, and Oceania | East Asia | Low-income | 8.8 | 6.4 | 11.2 |
| Djibouti | Sub‐Saharan Africa | Eastern Sub‐Saharan Africa | Lower-middle-income | 8.9 | 6.6 | 11.2 |
| Dominica | Latin America and the Caribbean | Caribbean | Upper-middle-income | 9.7 | 7.0 | 12.3 |
| Dominican Republic | Latin America and the Caribbean | Caribbean | Upper-middle-income | 10.8 | 6.8 | 14.8 |
| Ecuador | Latin America and the Caribbean | Andean Latin America | Upper-middle-income | 10.5 | 5.7 | 15.2 |
| Egypt | North Africa and the Middle East | North Africa and the Middle East | Lower-middle-income | 5.6 | 1.3 | 9.9 |
| El Salvador | Latin America and the Caribbean | Central Latin America | Lower-middle-income | 9.3 | 6.7 | 11.9 |
| Equatorial Guinea | Sub‐Saharan Africa | Central Sub‐Saharan Africa | Upper-middle-income | 7.2 | 4.6 | 9.8 |
| Eritrea | Sub‐Saharan Africa | Eastern Sub‐Saharan Africa | Low-income | 8.1 | 5.5 | 10.6 |
| Eswatini | Sub‐Saharan Africa | Southern Sub‐Saharan Africa | Lower-middle-income | 7.1 | 3.8 | 10.5 |
| Ethiopia | Sub‐Saharan Africa | Eastern Sub‐Saharan Africa | Low-income | 6.6 | 3.7 | 9.6 |
| Fiji | Southeast Asia, East Asia, and Oceania | Oceania | Upper-middle-income | 6.3 | 2.0 | 10.7 |
| Gabon | Sub‐Saharan Africa | Central Sub‐Saharan Africa | Upper-middle-income | 5.7 | 0.6 | 10.9 |
| Georgia | Central Europe, Eastern Europe, and Central Asia | Central Asia | Upper-middle-income | 11.7 | 8.4 | 15.0 |
| Ghana | Sub‐Saharan Africa | Western Sub‐Saharan Africa | Lower-middle-income | 5.9 | 2.4 | 9.5 |
| Grenada | Latin America and the Caribbean | Caribbean | Upper-middle-income | 9.4 | 6.8 | 12.0 |
| Guatemala | Latin America and the Caribbean | Central Latin America | Upper-middle-income | 11.3 | 7.3 | 15.3 |
| Guinea | Sub‐Saharan Africa | Western Sub‐Saharan Africa | Low-income | 9.3 | 5.9 | 12.6 |
| Guinea Bissau | Sub‐Saharan Africa | Western Sub‐Saharan Africa | Low-income | 6.7 | 4.5 | 9.0 |
| Guyana | Latin America and the Caribbean | Caribbean | Upper-middle-income | 6.6 | 2.0 | 11.2 |
| Haiti | Latin America and the Caribbean | Caribbean | Low-income | 11.2 | 7.0 | 15.4 |
| Honduras | Latin America and the Caribbean | Central Latin America | Lower-middle-income | 9.0 | 4.8 | 13.3 |
| India | South Asia | South Asia | Lower-middle-income | 7.9 | 5.1 | 10.8 |
| Indonesia | Southeast Asia, East Asia, and Oceania | Southeast Asia | Lower-middle-income | 8.3 | 6.0 | 10.7 |
| Iran | North Africa and the Middle East | North Africa and the Middle East | Upper-middle-income | 8.5 | 5.8 | 11.1 |
| Iraq | North Africa and the Middle East | North Africa and the Middle East | Upper-middle-income | 5.7 | 2.4 | 9.0 |
| Jamaica | Latin America and the Caribbean | Caribbean | Upper-middle-income | 9.5 | 6.5 | 12.5 |
| Jordan | North Africa and the Middle East | North Africa and the Middle East | Upper-middle-income | 10.1 | 3.6 | 16.5 |
| Kazakhstan | Central Europe, Eastern Europe, and Central Asia | Central Asia | Upper-middle-income | 8.8 | 3.2 | 14.4 |
| Kenya | Sub‐Saharan Africa | Eastern Sub‐Saharan Africa | Lower-middle-income | 6.5 | 2.4 | 10.6 |
| Kiribati | Southeast Asia, East Asia, and Oceania | Oceania | Lower-middle-income | 3.7 | -2.5 | 10.0 |
| Kosovo | Central Europe, Eastern Europe, and Central Asia | Central Europe | Upper-middle-income |  |  |  |
| Kyrgyz Republic | Central Europe, Eastern Europe, and Central Asia | Central Asia | Lower-middle-income | 6.7 | 2.0 | 11.4 |
| Lao | Southeast Asia, East Asia, and Oceania | Southeast Asia | Lower-middle-income | 7.9 | 5.6 | 10.3 |
| Lebanon | North Africa and the Middle East | North Africa and the Middle East | Upper-middle-income | 8.4 | 5.9 | 10.8 |
| Lesotho | Sub‐Saharan Africa | Southern Sub‐Saharan Africa | Lower-middle-income | 11.1 | -2.9 | 25.1 |
| Liberia | Sub‐Saharan Africa | Western Sub‐Saharan Africa | Low-income | 8.3 | 4.3 | 12.2 |
| Libya | North Africa and the Middle East | North Africa and the Middle East | Upper-middle-income | 7.4 | 4.5 | 10.2 |
| Madagascar | Sub‐Saharan Africa | Eastern Sub‐Saharan Africa | Low-income | 7.0 | 3.5 | 10.6 |
| Malawi | Sub‐Saharan Africa | Eastern Sub‐Saharan Africa | Low-income | 8.2 | 4.8 | 11.7 |
| Malaysia | Southeast Asia, East Asia, and Oceania | Southeast Asia | Upper-middle-income | 8.5 | 5.5 | 11.5 |
| Maldives | Southeast Asia, East Asia, and Oceania | Southeast Asia | Upper-middle-income | 10.9 | 5.6 | 16.1 |
| Mali | Sub‐Saharan Africa | Western Sub‐Saharan Africa | Low-income | 5.6 | 2.5 | 8.6 |
| Marshall Islands | Southeast Asia, East Asia, and Oceania | Oceania | Upper-middle-income | 5.5 | 0.2 | 10.8 |
| Mauritania | Sub‐Saharan Africa | Western Sub‐Saharan Africa | Lower-middle-income | 7.9 | 2.9 | 12.9 |
| Mauritius | Southeast Asia, East Asia, and Oceania | Southeast Asia | Upper-middle-income | 9.0 | 6.1 | 11.8 |
| Mexico | Latin America and the Caribbean | Central Latin America | Upper-middle-income | 12.1 | 5.8 | 18.4 |
| Micronesia | Southeast Asia, East Asia, and Oceania | Oceania | Lower-middle-income | 4.9 | -0.9 | 10.8 |
| Moldova | Central Europe, Eastern Europe, and Central Asia | Eastern Europe | Lower-middle-income | 9.6 | 5.6 | 13.6 |
| Mongolia | Central Europe, Eastern Europe, and Central Asia | Central Asia | Lower-middle-income | 11.0 | 7.5 | 14.4 |
| Montenegro | Central Europe, Eastern Europe, and Central Asia | Central Europe | Upper-middle-income | 12.9 | 9.5 | 16.2 |
| Morocco | North Africa and the Middle East | North Africa and the Middle East | Lower-middle-income | 6.9 | 3.1 | 10.8 |
| Mozambique | Sub‐Saharan Africa | Eastern Sub‐Saharan Africa | Low-income | 6.1 | 1.2 | 10.9 |
| Myanmar | Southeast Asia, East Asia, and Oceania | Southeast Asia | Lower-middle-income | 7.9 | 4.5 | 11.2 |
| Namibia | Sub‐Saharan Africa | Southern Sub‐Saharan Africa | Upper-middle-income | 6.6 | 2.4 | 10.9 |
| Nauru | Southeast Asia, East Asia, and Oceania | Oceania | Upper-middle-income | 4.2 | -2.6 | 10.9 |
| Nepal | South Asia | South Asia | Low-income | 7.7 | 3.8 | 11.6 |
| Nicaragua | Latin America and the Caribbean | Central Latin America | Lower-middle-income | 6.3 | 2.1 | 10.5 |
| Niger | Sub‐Saharan Africa | Western Sub‐Saharan Africa | Low-income | 5.9 | 2.7 | 9.0 |
| Nigeria | Sub‐Saharan Africa | Western Sub‐Saharan Africa | Lower-middle-income | 7.4 | 3.2 | 11.6 |
| North Macedonia | Central Europe, Eastern Europe, and Central Asia | Central Europe | Upper-middle-income | 13.2 | 10.1 | 16.2 |
| Pakistan | South Asia | South Asia | Lower-middle-income | 5.6 | 2.0 | 9.2 |
| Papua New Guinea | Southeast Asia, East Asia, and Oceania | Oceania | Lower-middle-income | 6.0 | 2.7 | 9.4 |
| Paraguay | Latin America and the Caribbean | Tropical Latin America | Upper-middle-income | 9.2 | 6.9 | 11.5 |
| Peru | Latin America and the Caribbean | Andean Latin America | Upper-middle-income | 9.4 | 6.2 | 12.5 |
| Philippines | Southeast Asia, East Asia, and Oceania | Southeast Asia | Lower-middle-income | 7.6 | 5.2 | 10.0 |
| Romania | Central Europe, Eastern Europe, and Central Asia | Central Europe | Upper-middle-income | 12.9 | 9.3 | 16.5 |
| Russia | Central Europe, Eastern Europe, and Central Asia | Eastern Europe | Upper-middle-income | 13.2 | 9.8 | 16.6 |
| Rwanda | Sub‐Saharan Africa | Eastern Sub‐Saharan Africa | Low-income | 5.4 | 1.4 | 9.5 |
| Samoa | Southeast Asia, East Asia, and Oceania | Oceania | Upper-middle-income | 2.6 | -5.2 | 10.4 |
| Sao Tome and Principe | Sub‐Saharan Africa | Western Sub‐Saharan Africa | Lower-middle-income | 6.1 | 2.9 | 9.4 |
| Senegal | Sub‐Saharan Africa | Western Sub‐Saharan Africa | Lower-middle-income | 5.6 | 2.1 | 9.1 |
| Serbia | Central Europe, Eastern Europe, and Central Asia | Central Europe | Upper-middle-income | 13.4 | 10.1 | 16.7 |
| Sierra Leone | Sub‐Saharan Africa | Western Sub‐Saharan Africa | Low-income | 6.2 | 1.3 | 11.2 |
| Solomon Islands | Southeast Asia, East Asia, and Oceania | Oceania | Lower-middle-income | 4.7 | 0.6 | 8.9 |
| Somalia | Sub‐Saharan Africa | Eastern Sub‐Saharan Africa | Low-income | 3.8 | 1.1 | 6.4 |
| South Africa | Sub‐Saharan Africa | Southern Sub‐Saharan Africa | Upper-middle-income | 7.7 | 1.4 | 14.0 |
| South Sudan | Sub‐Saharan Africa | Eastern Sub‐Saharan Africa | Low-income |  |  |  |
| Sri Lanka | Southeast Asia, East Asia, and Oceania | Southeast Asia | Upper-middle-income | 8.6 | 6.2 | 10.9 |
| St Lucia | Latin America and the Caribbean | Caribbean | Upper-middle-income | 9.5 | 6.4 | 12.6 |
| St Vincent and the Grenadines | Latin America and the Caribbean | Caribbean | Upper-middle-income | 9.8 | 7.4 | 12.2 |
| State of Palestine | North Africa and the Middle East | North Africa and the Middle East | Lower-middle-income | 5.5 | 2.0 | 8.9 |
| Sudan | North Africa and the Middle East | North Africa and the Middle East | Lower-middle-income | 4.7 | 1.5 | 7.9 |
| Suriname | Latin America and the Caribbean | Caribbean | Upper-middle-income | 9.0 | 6.6 | 11.4 |
| Syria | North Africa and the Middle East | North Africa and the Middle East | Low-income | 5.4 | 1.3 | 9.6 |
| Tajikistan | Central Europe, Eastern Europe, and Central Asia | Central Asia | Low-income | 6.9 | 1.2 | 12.6 |
| Tanzania | Sub‐Saharan Africa | Eastern Sub‐Saharan Africa | Low-income | 5.3 | 2.3 | 8.2 |
| Thailand | Southeast Asia, East Asia, and Oceania | Southeast Asia | Upper-middle-income | 9.3 | 6.7 | 11.9 |
| The Gambia | Sub‐Saharan Africa | Western Sub‐Saharan Africa | Low-income | 8.3 | 3.7 | 13.0 |
| Timor-Leste | Southeast Asia, East Asia, and Oceania | Southeast Asia | Lower-middle-income | 7.5 | 4.4 | 10.5 |
| Togo | Sub‐Saharan Africa | Western Sub‐Saharan Africa | Low-income | 5.2 | 2.1 | 8.2 |
| Tonga | Southeast Asia, East Asia, and Oceania | Oceania | Upper-middle-income | 3.6 | -3.7 | 10.9 |
| Tunisia | North Africa and the Middle East | North Africa and the Middle East | Lower-middle-income | 8.2 | 5.6 | 10.7 |
| Turkey | North Africa and the Middle East | North Africa and the Middle East | Upper-middle-income | 6.8 | 2.1 | 11.5 |
| Turkmenistan | Central Europe, Eastern Europe, and Central Asia | Central Asia | Upper-middle-income | 11.6 | 7.9 | 15.2 |
| Tuvalu | Southeast Asia, East Asia, and Oceania | Oceania | Upper-middle-income | 4.5 | -1.6 | 10.6 |
| Uganda | Sub‐Saharan Africa | Eastern Sub‐Saharan Africa | Low-income | 6.8 | 3.6 | 10.0 |
| Ukraine | Central Europe, Eastern Europe, and Central Asia | Eastern Europe | Lower-middle-income | 13.0 | 10.1 | 15.9 |
| Uzbekistan | Central Europe, Eastern Europe, and Central Asia | Central Asia | Lower-middle-income | 7.8 | 2.6 | 13.0 |
| Vanuatu | Southeast Asia, East Asia, and Oceania | Oceania | Lower-middle-income | 5.2 | 1.3 | 9.1 |
| Venezuela | Latin America and the Caribbean | Central Latin America | Upper-middle-income | 10.3 | 7.7 | 12.8 |
| Vietnam | Southeast Asia, East Asia, and Oceania | Southeast Asia | Lower-middle-income | 9.2 | 6.8 | 11.7 |
| Yemen | North Africa and the Middle East | North Africa and the Middle East | Low-income | 5.1 | 0.7 | 9.6 |
| Zambia | Sub‐Saharan Africa | Eastern Sub‐Saharan Africa | Lower-middle-income | 8.2 | 5.2 | 11.3 |
| Zimbabwe | Sub‐Saharan Africa | Southern Sub‐Saharan Africa | Lower-middle-income | 6.8 | 3.7 | 10.0 |

Note: estimates for South Sudan and Kosovo could not be computed due to the lack of 2020 data.
