# Supplementary material for: Gestational weight gain at the national, regional, and income group levels based on 234 national household surveys from 70 low-income and middle-income countries
Source: PLOS Glob Public Health. 2024 Sep 4;4(9):e0003484. doi: 10.1371/journal.pgph.0003484 (PMC11373806; doi:10.1371/journal.pgph.0003484)
Supplement: Attachment — Submitted filename: GWG_survey_data_cover_letter_v3 - Copy.docx [file pgph.0003484.s004.docx]

PGPH-D-24-00785
Gestational weight gain at the national, regional, and income group levels based on 234 national household surveys from 70 low-income and middle-income countries
PLOS Global Public Health

Dear Dr. Petancio,

We thank you for the careful assessment of our manuscript. The “Data Availability statement” has been corrected, and the updated files (clean and marked) have been attached to the submission system.

I hope the correction meets the journal criteria.

Best,

Janaína Calu Costa

On behalf of all authors
